# Treatment outcomes and HIV drug resistance of patients switching to second-line regimens after long-term first-line antiretroviral therapy: An observational cohort study

**DOI:** 10.1097/MD.0000000000011463

**Published:** 2018-07-13

**Authors:** Pi Cao, Bin Su, Jianjun Wu, Zhe Wang, Jiangzhou Yan, Chang Song, Yuhua Ruan, Hui Xing, Yiming Shao, Lingjie Liao

**Affiliations:** a State Key Laboratory of Infectious Disease Prevention and Control, National Center for AIDS/STD Control and Prevention, Chinese Center for Disease Control and Prevention, Collaborative Innovation Center for Diagnosis and Treatment of Infectious Diseases, Beijing; b Anhui Center for Disease Control and Prevention, Hefei, Anhui; c Henan Center for Disease Control and Prevention, Zhenzhou, Henan, China.

**Keywords:** CD4 cell count, China, human immunodeficiency virus, second-line regimen, viral load

## Abstract

To investigate the responses to switching to second-line regimens among patients who had received a long-term first-line antiretroviral therapy.

Patients switching to second-line regimens from June 2008 to June 2015 were enrolled from an observational cohort. In addition, patients continuing first-line therapy and had a viral load <1000 copies/mL were included as controls in July 2012. All these patients were followed-up for 36 months or until June 2016. The virological, immunological outcomes, and drug resistance were evaluated. Virological failure was defined as viral load ≥1000 copies/mL after 6 months of treatment since the start of the study.

There were 304 patients switching to second-line regimens and 46 patients remaining on first-line therapy enrolled while having received first-line therapy for a median of 7.6 years. Patients with plasma viral load (VL) ≥1000 copies/mL before switching to second-line regimens had a sharp decline in the proportion of virological failure with 26.7%, 20.4%, and 17.0% at 12, 24, and 36 months after regimen switch, respectively (trend test, *P* < .001). Among these patients, individuals with drug resistance (DR) had a better virological responses as compared with those without DR after regimen switching. While patients with VL <1000 copies/mL at inclusion remained a high rate of viral suppression after switching to second-line regimens. So did patients continuing first-line therapy. Among patients with VL ≥1000 copies/mL before switching to second-line regimens, the rates of drug resistance were decreased from 79.4% at inclusion to 7.5% at 36 months of regimen switch, with the proportion of NRTI- and NNRTI-related drug resistance from 67.2% and 79.4% to 5.4% and 7.5%, respectively. No PI-related resistance was found. Having self-reported missing doses within a month at follow-ups were independently associated with virological failure at 36 months of switching.

HIV-infected patients had viral load ≥1000 copies/mL at regimen switch after a long duration of first-line therapy had good virological responses to second-line regimens, especially those harbored drug resistant variants at regimen switch. However, patients with suppressive first-line therapy did not appear to benefit virologically from switching to second-line regimens.

## Abstract 2

Supplemental Digital Content is available in the text

## 1 Introduction

In the past 2 decades, substantial efforts have been devoted to introducing and scaling antiretroviral therapy (ART).^[1]^ It was reported that 19.5 million people living with human immunodeficiency virus (HIV) were receiving antiretroviral therapy (ART) globally by the end of 2016.^[2]^ With more patients on ART, first-line treatment failure is increasing, and the need to second-line switch is growing.^[3–6]^ It was estimated that in 2015, 3.5 million people living with HIV in low- and middle-income countries would switch to second-line treatment.^[4]^

Like other resource-limited countries, China started National Free Antiretroviral Treatment Program in early 2000s.^[7,8]^ The program was firstly began in former plasma donors who acquired HIV infection in the mid-1990s through unhygienic blood and plasma donation, and then expanded to other HIV-infected populations. According to the recommendation of the World Health Organization (WHO), the first-line regimens consisted of 2 nucleoside reverse transcriptase inhibitors (NRTIs) and 1 non-nucleoside reverse transcriptase inhibitor (NNRTI), and were stavudine (d4T) or azidothymidine (AZT), didanosine (ddI), plus nevirapine (NVP), or efavirenz (EFV) before 2005. In later years ddI and d4T were replaced by lamivudine (3TC) and tenofovir (TDF), respectively.^[9]^ There was a high prevalence of acquired HIV drug resistance (DR) and treatment failure among patients who start ART with ddI-based first line regimens, and also among those with long-term of ART.^[10–13]^

In 2008 China initiated second-line treatment, of which the regimens consists ritonavir-boosted lopinavir (LPV/r) and 2 other NRTIs, and the number of patients receiving second-line treatment is growing.^[7]^ Previous study reported a high rate of virological failure after switching to second-line regimens.^[14]^ However, other studies indicate a high level of viral suppression among HIV-infected patients switched to second-line ART.^[15,16]^ However, there were few studies on evaluating the efficacy of long-term second-line therapy in China.^[9,17]^ The objective of this study was to assess treatment outcomes of 36-month switching to second-line ART among Chinese patients who had been on first-line therapy for a long duration in an observational cohort.

## 2 Materials and methods

### 2.1 Study design and study population

Patients switching to second-line therapy from June 2008 to June 2015 were enrolled from an observational cohort, which was established in rural areas of Henan and Anhui provinces in China, as described previously.^[18]^ Inclusion criteria of this study were: 18 years or older, having been on receiving first-line therapy for at least 2.5 years before switching to second-line therapy, and having plasma viral load, CD4 cell count, and drug resistance genotyping within 6 months before the switch, and willing to provide informed consent. Additional patients who remained on first-line therapy and had VL <1000 copies/mL in June 2012 (the median date of switch to second-line regimens in patients firstly mentioned) and had not any change of regimen components thereafter were included in parallel. All the patients were followed-up for 36 months of switching to second-line regimens or continuing first-line therapy, death or up to May 2016. The institutional review board at the National Center for AIDS/STD Control and Prevention, Chinese Center for Disease Control and Prevention approved this study and experimental protocols.

### 2.2 Laboratory tests

Blood sample were sent to the laboratory of NCAIDS in Beijing immediately after collected, and then CD4 cell count, viral load, and HIVDR (HIV drug resistance) genotyping were performed. CD4 cell count was measured using flow cytometry (FACS Calibur, BD Company, Franklin Lakes, NJ) within 2 hours after sample arriving. Meanwhile, plasma was separated by centrifugation and instantly stored at −80 °C for testing viral load and drug resistance. Plasma HIV RNA was quantified with real-time NASBA (NucliSense Easy Q, bioMerieur, France) or COBAS (Roche Applied Scence, Germany).^[19]^ For samples with plasma viral loads (VL) ≥1000 copies/mL, HIVDR genotyping was carried out by an in-house Polymerase Chain Reaction protocol.^[19]^ HIV drug resistance was determined according to the Stanford University HIV Drug Resistance Database (https://hivdb.stanford.edu/hivdb/by-sequences/), and were defined by all drug resistance mutations, including low-, intermediate-, or high-level resistance.^[20]^

### 2.3 Data analysis

Virological failure (VF) was defined as VL ≥1000 copies/mL after 6 months of treatment from the start of study. Cochran-Armitage Test for Trend was used to evaluate the rate of virological failure and drug resistance at different time point. Repeated-Measures Analysis of Variance was used to evaluate immunological response (CD4 cell count) at inclusion and 12, 24, and 36 months after switched to second-line regimens. For virological outcomes between groups, we used repeated measures for categorical data to analyze difference. Factors associated with viral load ≥1000 copies/mL at 36 months of second-line therapy were analyzed using logistic regression model, and covariates with *P* < .1 in univariate analyses were entered in multivariate analyses. Statistical significance was accepted at *P* < .05 for 2-side tests. SAS V9.4 (SAS Institute Inc., Cary, NC) was used for all statistical analyses.

## 3 Results

### 3.1 Characteristics of study population

By June 2015, 322 patients switching to second-line regimens were enrolled from an observational cohort established in rural areas of Central China. The mid-point regimen switch time was June 2012. Of these patients, 18 were removed, including 7 patients who died and 11 stop ART within 6 months after switching to second-line regimens. Finally, 304 patients switched to second-line regimens were enrolled in our study, and baseline was considered the first day of second-line ART, information of VL and CD4 cell count at baseline were from the latest follow-up within 6 months before switching to second-line regimens. Meanwhile, 46 patients continuing first line therapy without any change of regimens components since June 2012 and had a plasma viral load <1000 copies/mL at that time were included as the first-line group. For these patients, baseline was considered the date of June 16th, 2012 (median date of switching second-line regimens). As the start date of second-line therapy varied and few patients occasionally missed yearly visit, the number of patients differed at time points of 12, 24, and 36 months, with 350, 312, and 261 patients being followed up (Table 1), respectively. By June 2016, there were 5 and 17 patients on first- and second-line therapy died, with the mortality rates of 4.2 and 2.1 per 100 person-years, respectively.

**Table 1:**
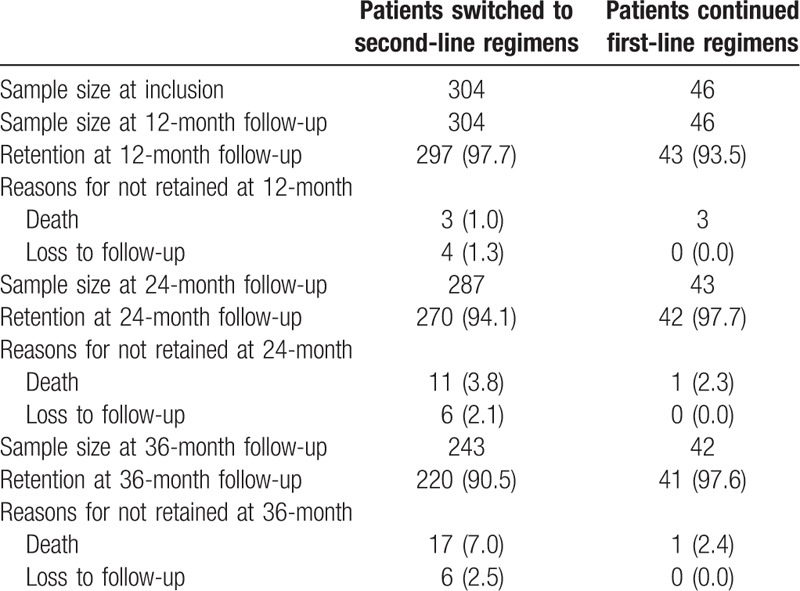
Study flow chart.

Of the 350 patients, the median age was 47 years old (interquartile [IQR] 43–52). The majority were women (61.1%), former plasma donors (97.7%), married or living with partner (87.7%), poorly educated with 73.4% only received education in primary school or being illiterate (Table 2). Most of the subjects start ART with ddI-based first line regimens (78.8%) in 2004, having been on first-line ART for 7.6 years (IQR 6.0–8.8). The recorded reasons of switching to second-line regimens were ART failure (78.3%), followed by side effects (7.6%), drug interactions (6.2%), and unknown reasons. In 304 patients switching to second-line therapy, 180 (59.2%) had plasma viral load ≥1000 copies/mL, and 124 (40.8%) had plasma viral load <1000 copies/mL (baseline). For these 304 patients, regimens used were 3TC + TDF + LPV/r (303 patients), and 3TC + AZT + LPV/r (1 patient). In contrast, all patients continuing first-line therapy had VL <1000 copies/mL at inclusion. Among these patients, regimens used were AZT/D4T + 3TC + NVP/EFV (44 patients), and 3TC + TDF + NVP/EFV (2 patients).

**Table 2:**
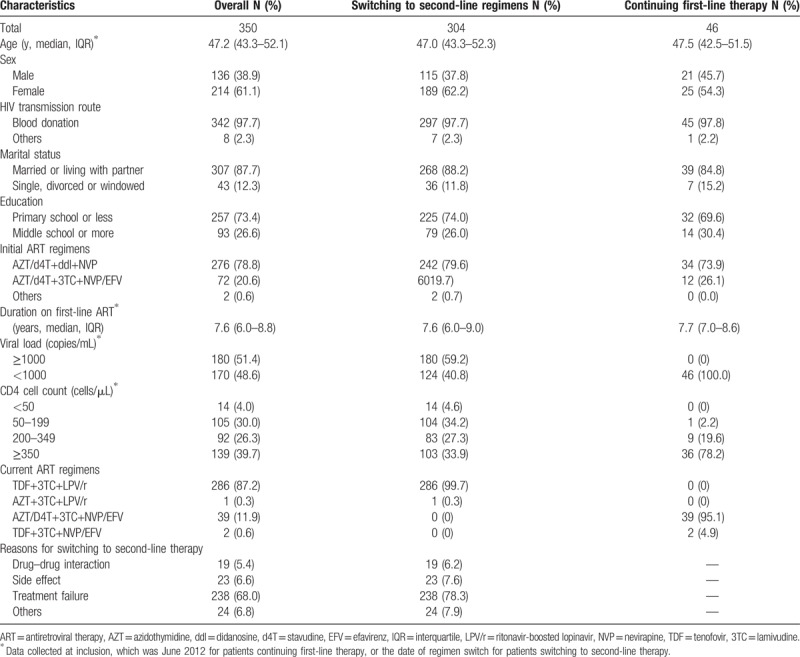
Characteristics of patients switching to second-line regimens or continuing first-line therapy.

### 3.2 Measurements of plasma viral load

**Figure 1:**
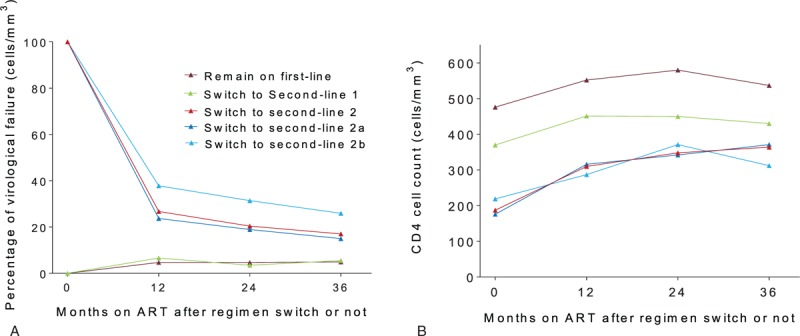
The rate of VF and CD4 cell count among patients switched before and 12, 24, and 36 months of second-line therapy. Remain on first-line (brown): patients continuing first line therapy; switch to second-line 1 (green): patients with viral load <1000 copies/mL before switching to second-line regimens; switch to second-line 2 (red): patients with viral load ≥1000 copies/mL before switching to second-line regimens; switch to second-line 2a (dark blue); patients with viral load ≥1000 copies/mL and drug resistance before switching to second-line regimens; switch to second-line 2b (light blue): patients with viral load ≥1000 copies/mL and not drug resistance before switching to second-line regimens.

In 304 patients switching to second-line therapy, the proportions of VL ≥1000 copies/mL were decreased dramatically from 59.2% to 18.5% in the first year of switch, the trend was prolonged during the second and third year of switch, with 14.1% and 13.2% of VF, respectively (trend test, *P* < .001). The decline trend in VF was more pronounced in 180 patients who had VL ≥1000 copies/mL before switching to second-line ART, with proportions of 26.7% (47/176), 20.4% (34/167), and 17.0% (25/147) at 1, 2, and 3 years of switch, respectively (trend test, *P* < .001). Of these 180 patient, 143 (79.4%) had drug resistance variant at the switch. It is notable that these patients descended more sharply in the rates of VF compared with patients without drug resistance (*P* = .03). Other 124 patients who had VL <1000 copies/mL at regimen switch had stable virological suppression rates of around 95% from 1 to 3 years of the switch, which was similar to the virological suppression rates among patients remained on first-line therapy (Fig. 1).

### 3.3 CD4 cell count outcomes

Among 304 patients switching to second-line regimens, a significant raise in CD4 cell count was found while on second-line therapy, from 262 (IQR 139–410) cells/μL at baseline, to 343.7 (IQR 226–503) cells/μL, 394 (IQR 248–555) cells/μL, and 380 (IQR 230–554) cells/μL after 12, 24, and 36 months of switch (*P* = .01), respectively. Among these patients, 180 patients who had VL ≥1000 copies/mL at baseline had a marked increase of CD4 cell count from 187 (IQR 997–3097) cells/μL at baseline to 310 (IQR 189–430), 348 (IQR 212–513), 364 (IQR 218–487), after 12, 24, and 36 months’ switch, respectively. Median increment of CD4 cell count were 64, 115, and 172 cells/μL during the first, the second, and the third years of regimen switch, respectively. Among patients with VL <1000 copies/mL at baseline, the number of CD4 cell count increase of 42 cells/μL from baseline to 12 months’ switch. In patients continue on first-line therapy, there was an increase of 78 cells/μL from baseline to first time point after June 16th, 2012.

### 3.4 HIV drug resistance

In patients with VF at regimen switch, the prevalence of HIV drug resistance declined from 79.4% at baseline to 7.5% after 36 months of second-line therapy (Table 3). The patients with VF and with drug resistance had much higher rates of drug resistance at 12, 24, and 36 months of switching to second-line regimens than those without drug resistance at baseline (*P* < .001 at 12, 24, and 36 months). For patients switched to second-line regimens with VL <1000 copies/mL and patients continued first-line regimens, the rates of drug resistance were stable at around 2%.

**Table 3:**
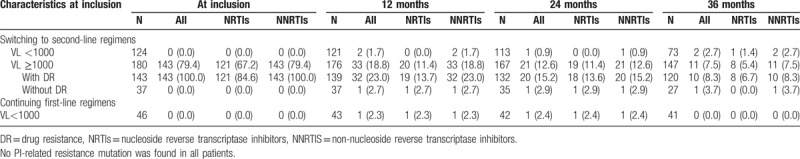
HIV drug resistance at 12, 24, and 36 months of switching to second-line regimens or continuing first-line therapy.

Among 143 patients with VF and drug resistance before switching to second-line regimens, the rates of NNRTI- and NRTI-related drug resistance mutations were from 100% and 84.6% at baseline, to 8.3% and 6.7% after 36 months, respectively. No protease inhibitor (PI)-related resistance mutation was found before and after second-line switch. At baseline, the most common NNRTI-related mutations included K103NS (57.3%), Y181C (48.3%), G190AS (32.2%), and the most frequent NRTI-related resistance mutations were T215CFYI (58.0%), M184V mutations (54.5%), M41L mutations (49.7%), and L210W mutations (32.9%). After 36 months of second-line therapy, the most common NNRTI-related mutations included K103NS (8.3%), Y181C (5.0%), G190AS (2.5%), and the most frequent NRTI-related resistance mutations were T215CFYI (5.8%), M184V mutations (5.0%), M41L mutations (4.2%), and T69N mutations (3.3%). Details are shown in Supplemental Digital Contents.

### 3.5 Factors associated with virological failure at 36 months of switching to second-line regimens

To investigate factors associated with VF at 36 months of switching to second-line therapy, we used logistic regression models among patients with VF at baseline. One hundred forty-seven patients who had been on second-line therapy for 36 months were included in the analysis (Table 4). Both in univariate and multivariable analysis, having self-report missed doses within a month at follow-ups before switch to second-line regimens (odds ratio [OR] = 3.41, 95% Confidence interval [CI]: 1.39–8.35, *P* = .01) was associated with viral load ≥1000 copies/mL at 36 months of regimen switch.

**Table 4:**
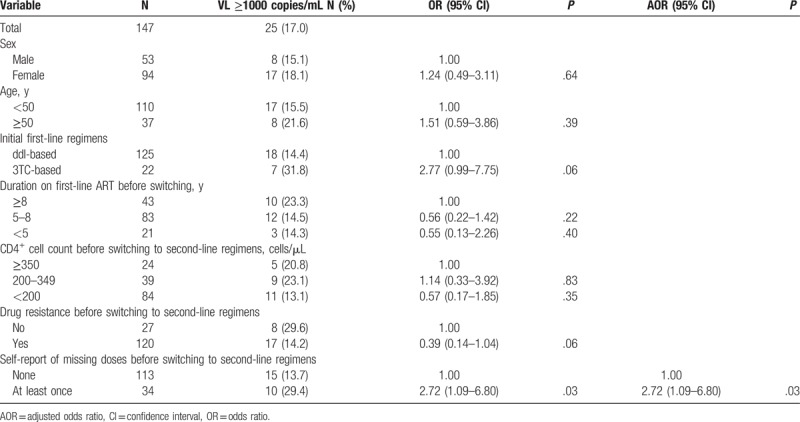
Factors associated with virological failure at 36 months of switching to second-line regimens among patients with viral load ≥1000 copies/mL at regimen switch.

## 4 Discussion

In this study, we investigated the effect of 36-month switching to second-line regimens after median 7.6 years of first-line treatment. The rates of virological failure among patients with VL ≥1000 copies/mL at baseline declined rapidly after switching to second-line regimens. It is consistent with our previous study with a shorter duration (12 months) of switching to second-line therapy, and other recent studies.^[9,17,21]^ In the study among Sub-Saharan African patients failing first-line treatment, the VF rates after switching to second-line therapy went down along time, with 14.6%, 15.2%, and 11.1% at 12, 24, 36 months, respectively.^[21]^ In another study among Chinese patients, the virological suppression rates (<400 copies/mL) went up to 90% after 120 weeks of switching to second-line regimens.^[17]^ However, in a meta-analysis of retrospective second-line outcomes in low-and middle-income countries, the proportion of HIV-infected patients failing therapy was 23.1%, 26.7%, and 38.0% at 12, 24, and 36 months, and poor adherence appeared to be the main driver of virological failure.^[14]^

Regarding virological response and the rate of HIV drug resistance, there were no significant difference between patients switching to second-line therapy and those continuing first-line therapy while having VL <1000 copies/mL at baseline. Boettiger et al^[22]^ warned that early switching might lead to quicker exhaustion of treatment options. However, in the real settings, patients might be inclined to second-line therapy, as they believed it was better than first-line therapy.^[9]^ Large scaled study is necessary to investigate the treatment outcome among patients with early switch to second-line therapy.

Our study also shows that among patients with first-line virological failure at regimen switch, individuals who had drug resistance variants had a higher rate of viral suppression than those with no detectable HIV drug resistance. Consistently, Hosseinipour et al^[23,24]^ showed that high rates of virological suppression and good immunological recovery achieved after second-line switch despite of extensive baseline drug resistance. We hypothesized that having drug resistance might be an indicator of a better adherence, when compared with patients of VF but not detectable drug resistance. A recent study had also shown that a less proportion of patients with baseline drug resistance had subtherapeutic antiretroviral drug concentration.^[25]^ However, we did not find a better self-report adherence in patients who had baseline drug resistance. To confirm the hypothesis, more researches should be performed on the relationship of adherence and drug resistance among patients with virological failure. Furthermore, drug resistance mutations such as M184V and TAMs in RT region may compromise HIV replicative fitness and lead to a lower viral load.^[26]^

In our study, patients having missed doses within a month at follow-ups before switching to second-line regimens are more likely to with VF, this again show that poor adherence is a major risk factor for second-line treatment. It is notable that there was a significant improvement of self-report adherence after switch (data not shown). Other factors may have impacts on the effect of switch to second-line ART. It was shown that HLA B∗5701 positive patients had a high rate of viral suppression than those negative. HLA-B∗35 alleles was associated with post treatment control.^[27]^ Some other host factors such as SAMHD1 may also affect the outcome of ART.^[28]^ In this study, these host factors could not be taken into account as we did not have such data, but they are worth being further studied on their roles of ART outcome.

All patients in this study were infected with Thai-B HIV-1 strains. The prevalence of drug resistance persistently declined with the decrease of VF rate while on second-line therapy. Analyzing the results of this study, we found NNRTI-related mutations including K103NS, Y181C, G190AS, and K101E existed until 36 months of second-line ART, which might reflect a low impact on replicative fitness of such class of mutations.^[29]^ It is not exceptional that NRTI-related drug resistance mutations such as M184V, and TAMs lasting for 36 months, as the backbone of second-line regimens is still NRTI-class of antiretroviral drugs. There was no PI-related mutation found during the observation, which is similar to results of some studies, but not to others.^[30,31]^ It would take a longer time to generate PI-related resistance mutations, as ritonavir (RTV)-boosted protease inhibitor had a higher genetic barrier.^[32,33]^

As for immunological response to second-line therapy, CD4 cell count significantly increased across groups in this study. Similar findings showed that patient with VF at baseline had CD4 cell count raised from 157 cells/μL up to 307 cells/μL at week 120 of switching to second-line therapy.^[17]^ Other studies including our previous study also revealed a great immunological response after switching to second-line therapy.^[9,22,30,34,35]^ Suppressive ART might also have an impact on antibody responses to HIV infection.^[36,37]^ Some studies showed a weak antibody response in a proportion of patients with early ART and fewer patients starting ART in chronic HIV infection.^[38]^ In addition, patients with long-term suppressive ART still had continuous antigenic stimulation and lead to the evolution of HIV-specific antibody.^[37]^ In our study, patients with VF prior to switch might have different HIV-specific antibody responses compared with patients without VF, further study should focus on the trend of antibody responses to HIV and give some hints to HIV incidence surveillance and vaccine development.^[39,40]^

In our study, approximate 20% of the patients had virological failure even after switching to second-line treatment. Besides introducing more third-line antiretroviral drugs to China, comprehensive adherence supports such as adherence re-education, more prompt and frequent viral load monitoring, psychological counseling, and less social discrimination would help these patients achieve virological suppression.^[41,42]^

There are several limitations. Firstly, patients were not randomly assigned as an observational study; in addition, the majority of the subjects were HIV infected FPDs, although there was no evidence of different ART adherence between FPDs and other patients; cautions should be taken to extrapolate the results to the general patients. Secondly, the initial regimens of most patients were early ddI-based but not the first-line regimens currently recommended by the WHO. However, all these patients changed to 3TC-based regimens early or later after 2005, and had remained on the latter regimens for at least 1 year (median 4.8 years). Our data also showed no significant difference in rate of VF after 36 months of second-line therapy between patients with ddI- and 3TC-based first line regimens.

In conclusion, our study showed patients had a significant decrease in VF rate throughout 36 months of second-line switch. The decline trends were remarkable in patients with virological failure at switch, and more pronounced in patients with drug resistance simultaneous. It is not suggested that patients on suppressive first-line ART switch to second-line regimens.

## Acknowledgments

The authors thank all of the study patients in our study and the staff from local CDCs for sample collection and assistance.

## Author contributions

**Conceptualization:** Yuhua Ruan, Hui Xing, Yiming Shao, Lingjie Liao.

**Data curation:** Pi Cao.

**Formal analysis:** Pi Cao.

**Investigation:** Pi Cao, Bin Su, Jianjun Wu, Zhe Wang, Jiangzhou Yan.

**Methodology:** Pi Cao, Jianjun Wu, Chang Song.

**Project administration:** Bin Su, Zhe Wang, Hui Xing, Yiming Shao, Lingjie Liao.

**Software:** Yuhua Ruan.

**Supervision:** Yuhua Ruan, Hui Xing, Yiming Shao, Lingjie Liao.

**Writing – original draft:** Pi Cao.

**Writing – review and editing:** Lingjie Liao.

## Supplementary Material

Supplemental Digital Content
