# Supplementary material for: Treatment outcomes and HIV drug resistance of patients switching to second-line regimens after long-term first-line antiretroviral therapy: An observational cohort study
Source: Medicine (Baltimore). 2018 Jul 13;97(28):e11463. doi: 10.1097/MD.0000000000011463 (PMC6076136; doi:10.1097/MD.0000000000011463)
Supplement: Supplemental Digital Content [file medi-97-e11463-s001.pdf]

**Treatment outcomes and HIV drug resistance of patients switching to second-line regimens after long-term first-line antiretroviral therapy**

Pi Cao, BS[1], Bin Su, MD[2], Jianjun Wu, MD[2], Zhe Wang, MD[3], Jiangzhou Yan, MD[3], Chang Song, BS[1], Yuhua Ruan, PhD[1], Hui Xing, PhD[1], Yiming Shao, PhD[1], Lingjie Liao, PhD[1]

Supplemental Digital Content 1 HIV drug resistance mutations among patients switched to second-line regimens viral load ≥1000 copies/ml and with drug resistance at inclusion

| | Before switching to second-line regimens N (%) | 12 months after switching to second-line regimens N (%) | 24 months after switching to second-line regimens N (%) | 36 months after switching to second-line regimens N (%) |
|---|---|---|---|---|
| Total | 143 | 139 | 132 | 120 |
| All | 143(100.0) | 32(23.0) | 20(15.2) | 10(8.3) |
| NRTIs | 121(84.6) | 19(13.7) | 18(13.6) | 8(6.7) |
| T215CFYI | 83(58.0) | 11(7.9) | 12(9.1) | 7(5.8) |
| M184V | 78(54.5) | 14(10.1) | 13(9.8) | 6(5.0) |
| M41L | 71(49.7) | 7(5.0) | 8(6.1) | 5(4.2) |
| L210W | 47(32.9) | 2(1.4) | 4(3.0) | 3(2.5) |
| K219EQ | 32(22.4) | 3(2.2) | 6(4.5) | 1(0.8) |
| D67NG | 30(21.0) | 2(1.4) | 7(5.3) | 3(2.5) |
| T69N | 32(22.4) | 2(1.4) | 4(3.0) | 4(3.3) |
| K70R | 26(18.2) | 3(2.2) | 2(1.5) | 0(0.0) |
| L74SV | 16(11.2) | 3(2.2) | 1(0.8) | 1(0.8) |
| NNRTIs | 143(100.0) | 32(23.0) | 20(15.2) | 10(8.3) |
| K103NS | 82(57.3) | 22(15.8) | 15(11.4) | 10(8.3) |
| Y181C | 69(48.3) | 9(6.5) | 9(6.8) | 6(5.0) |
| G190AS | 46(32.2) | 7(5.0) | 8(6.1) | 3(2.5) |
| V108I | 29(20.3) | 5(3.6) | 5(3.8) | 2(1.7) |
| K101E | 20(14.0) | 4(2.9) | 3(2.3) | 2(1.7) |
| E138GQ | 9(6.3) | 1(0.7) | 1(0.8) | 0(0.0) |
| Y188L | 6(4.2) | 0(0.0) | 0(0.0) | 0(0.0) |
| K238T | 5(3.5) | 1(0.7) | 1(0.8) | 0(0.0) |
| A98G | 4(2.8) | 1(0.7) | 0(0.0) | 0(0.0) |
| P225H | 1(0.7) | 1(0.7) | 0(0.0) | 0(0.0) |
| PIs | 0(0.0) | 0(0.0) | 0(0.0) | 0(0.0) |

**Treatment outcomes and HIV drug resistance of patients switching to second-line regimens after long-term first-line antiretroviral therapy**

Pi Cao, BS[1], Bin Su, MD[2], Jianjun Wu, MD[2], Zhe Wang, MD[3], Jiangzhou Yan, MD[3], Chang Song, BS[1], Yuhua Ruan, PhD[1], Hui Xing, PhD[1], Yiming Shao, PhD[1], Lingjie Liao, PhD[1]

Supplemental Digital Content 2 HIV drug resistance mutations among patients switched to second-line regimens with viral load ≥1000 copies/ml and without drug resistance at inclusion

| | Before switching to second-line regimens N (%) | 12 months after switching to second-line regimens N (%) | 24 months after switching to second-line regimens N (%) | 36 months after switching to second-line regimens N (%) |
|---|---|---|---|---|
| Total | 37 | 37 | 35 | 27 |
| All | 0(0.0) | 1(2.7) | 1(2.9) | 1(3.7) |
| NRTIs | 0(0.0) | 1(2.7) | 1(2.9) | 0(0.0) |
| M184V | 0(0.0) | 1(2.7) | 1(2.9) | 0(0.0) |
| NNRTIs | 0(0.0) | 1((2.7) | 1(2.9) | 1(3.7) |
| K103NS | 0(0.0) | 1(2.7) | 0(0.0) | 0(0.0) |
| E138GQ | 0(0.0) | 0(0.0) | 0(0.0) | 1(3.7) |
| Y188L | 0(0.0) | 0(0.0) | 1(2.9) | 0(0.0) |
| PIs | 0(0.0) | 0(0.0) | 0(0.0) | 0(0.0) |

**Treatment outcomes and HIV drug resistance of patients switching to second-line regimens after long-term first-line antiretroviral therapy**

Pi Cao, BS[1], Bin Su, MD[2], Jianjun Wu, MD[2], Zhe Wang, MD[3], Jiangzhou Yan, MD[3], Chang Song, BS[1], Yuhua Ruan, PhD [1], Hui Xing, PhD [1], Yiming Shao, PhD [1], Lingjie Liao, PhD [1]

Supplemental Digital Content 3 HIV drug resistance mutations among patients switched to second-line regimens with viral load <1000 copies/ml at inclusion

| | Before switching to second-line regimens N (%) | 12 months after switching to second-line regimens N (%) | 24 months after switching to second-line regimens N (%) | 36 months after switching to second-line regimens N (%) |
|---|---|---|---|---|
| Total | 124 | 121 | 113 | 73 |
| All | 0(0.0) | 2(1.7) | 1(0.9) | 2(2.7) |
| NRTIs | 0(0.0) | 0(0.0) | 0(0.0) | 1(1.4) |
| M184V | 0(0.0) | 0(0.0) | 0(0.0) | 1(1.4) |
| NNRTIs | 0(0.0) | 2(1.7) | 1(0.9) | 2(2.7) |
| K103NS | 0(0.0) | 1(0.8) | 0(0.0) | 1(1.4) |
| Y181C | 0(0.0) | 1(0.8) | 1(0.9) | 0(0.0) |
| G190AS | 0(0.0) | 0(0.0) | 0(0.0) | 1(1.4) |
| PIs | 0(0.0) | 0(0.0) | 0(0.0) | 0(0.0) |

**Treatment outcomes and HIV drug resistance of patients switching to second-line regimens after long-term first-line antiretroviral therapy**

Pi Cao, BS[1], Bin Su, MD[2], Jianjun Wu, MD[2], Zhe Wang, MD[3], Jiangzhou Yan, MD[3], Chang Song, BS[1], Yuhua Ruan, PhD[1], Hui Xing, PhD[1], Yiming Shao, PhD[1], Lingjie Liao, PhD[1]

Supplemental Digital Content 4 HIV drug resistance mutations among patients continuing first-line regimens

| | Before switching to second-line regimens N (%) | 12 months after switching to second-line regimens N (%) | 24 months after switching to second-line regimens N (%) | 36 months after switching to second-line regimens N (%) |
|---|---|---|---|---|
| Total | 46 | 43 | 42 | 41 |
| All | 0(0.0) | 1(2.3) | 1(2.4) | 0(0.0) |
| NRTIs | 0(0.0) | 1(2.3) | 1(2.4) | 0(0.0) |
| T215CFYI | 0(0.0) | 1(2.3) | 1(2.4) | 0(0.0) |
| M184V | 0(0.0) | 1(2.3) | 1(2.4) | 0(0.0) |
| M41L | 0(0.0) | 1(2.3) | 1(2.4) | 0(0.0) |
| L210W | 0(0.0) | 1(2.3) | 1(2.4) | 0(0.0) |
| NNRTIs | 0(0.0) | 1(2.3) | 1(2.4) | 0(0.0) |
| G190AS | 0(0.0) | 1(2.3) | 1(2.4) | 0(0.0) |
| K101E | 0(0.0) | 1(2.3) | 1(2.4) | 0(0.0) |
| A98G | 0(0.0) | 1(2.3) | 1(2.4) | 0(0.0) |
| PIs | 0(0.0) | 0(0.0) | 0(0.0) | 0(0.0) |

NNRTIs = non-nucleoside reverse transcriptase inhibitors, NRTIs = nucleoside reverse transcriptase inhibitors, PIs = protease inhibitors.
